# Methylphenidate is more effective to improve inhibitory control and working memory compared to tDCS in children and adolescents with attention deficit/hyperactivity disorder: a proof-of-concept study

**DOI:** 10.3389/fnins.2023.1170090

**Published:** 2023-07-07

**Authors:** Barbara D’Aiello, Giulia Lazzaro, Andrea Battisti, Pierpaolo Pani, Silvia Di Vara, Pietro De Rossi, Italo Pretelli, Floriana Costanzo, Stefano Vicari, Deny Menghini

**Affiliations:** ^1^Child and Adolescent Neuropsychiatry Unit, Bambino Gesù Children’s Hospital, IRCCS, Rome, Italy; ^2^Department of Human Science, LUMSA University, Rome, Italy; ^3^Department of Physiology and Pharmacology, Sapienza University, Rome, Italy; ^4^Department of Life Science and Public Health, Università Cattolica del Sacro Cuore, Rome, Italy

**Keywords:** MPH, drug treatments, non-invasive brain stimulation, transcranial direct current stimulation, executive functions, evidence-based medicine, neurodevelopmental disorders

## Abstract

**Introduction:**

Attention-deficit/hyperactivity disorder (ADHD) is characterized by an inappropriate, pervasive and persistent pattern of inattention, hyperactivity, and/or impulsivity and associated with substantial functional impairment. Despite considerable advances in the understanding and management of ADHD, some patients do not respond well to methylphenidate (MPH), the first-choice pharmacological treatment. Over the past decades, among non-invasive brain stimulation techniques, transcranial direct current stimulation (tDCS) has proven to be an effective and safe technique to improve behavior and cognition in children with neurodevelopmental disorders, including ADHD, by modifying cortical excitability. However, the effect of tDCS has never been directly compared with that of the MPH. The present randomized sham-controlled trial evaluated the effect of a single session of anodal tDCS compared with the administration of a single dose of MPH in children and adolescents with ADHD.

**Methods:**

After completing baseline assessment (T0), 26 children and adolescents with ADHD were exposed to 3 conditions with a 24-h interval-sessions: (A) a single session of anodal tDCS over the left dorsolateral prefrontal cortex (DLPFC); (B) a single session of sham tDCS over the left DLPFC; (C) a single dose of MPH.

**Results:**

Our results showed that after administering a single dose of MPH, children and adolescents with ADHD improved inhibitory control and visual–spatial WM compared with baseline, anodal, and sham tDCS. However, a single session of active tDCS over the left DLPFC was not effective compared with either baseline or sham tDCS.

**Discussion:**

In conclusion, our protocol in ADHD involving a single tDCS session did not demonstrate consistent improvements in neurocognitive features compared with baseline, sham tDCS, or single MPH administration. Different protocols need to be developed to further test the effectiveness of tDCS in improving ADHD symptoms.

## 1. Introduction

Attention-deficit/hyperactivity disorder (ADHD) is characterized by an inappropriate, pervasive and persistent pattern of severe inattention, hyperactivity, and/or impulsivity (American Psychiatric Association, 2013) with a worldwide prevalence of ~6–16% in children and adolescents (Danielson et al., 2022). ADHD develops during childhood, and persists into adulthood in most cases where it is associated with psychiatric comorbidities and poor quality of academic, social and professional life (Thomas et al., 2015).

Most people with ADHD have impairments in high-level cognitive functions necessary for goal-directed behaviors, such as inhibitory control, working memory (WM), and cognitive flexibility (Nigg and Casey, 2005; Sonuga-Barke et al., 2010; Willcutt, 2012). Neuroimaging studies have documented several abnormalities in the dorsolateral prefrontal cortex (DLPFC), orbital frontal cortex (OFC), anterior cingulate cortex, and basal ganglia (Cortese et al., 2012; McCarthy et al., 2014; Faraone et al., 2015; Norman et al., 2016; Lukito et al., 2020), probably due to dysfunction of dopaminergic and noradrenergic neurotransmission in the fronto-striatal pathway (Castellanos, 2002).

Methylphenidate (MPH) is the most commonly used drug for ADHD (Cortese et al., 2018). Although the specific mechanisms of action are not fully defined, MPH is thought to inhibit the protein responsible for dopamine reuptake into the synaptic space, DAT-1, thereby modulating the transmission of catecholaminergic neurotransmitters in fronto-striatal and fronto-parietal pathways (Arnsten, 2006; Schiffer et al., 2006; Volkow et al., 2007). Neuroimaging studies in both healthy individuals and patients with ADHD indicate that acute doses of MPH up-regulate and normalize brain regions known to be under-functioning in ADHD (Rubia et al., 2009).

More than 100 randomized placebo-controlled clinical trials ascertain that MPH is one of the most successful interventions for ADHD, reducing symptoms of inattention, hyperactivity, and impulsivity (Stein et al., 2003) as well as improving executive functions (Coghill et al., 2014; Guven et al., 2018). Nevertheless, about 30 percent of individuals do not respond well to MPH, have no long-term benefits, experience side effects (Faraone et al., 2015; Cortese et al., 2018) and, especially in adolescence, adhere poorly to treatment (Taylor et al., 2004). Moreover, non-pharmacological interventions such as cognitive-behavioral psychotherapy (Knouse et al., 2017), cognitive training (Scionti et al., 2020), parent training for preschool children (Rimestad et al., 2019) or dietary interventions (Li et al., 2020) exhibit small to moderate clinical effectiveness (Faraone et al., 2021). In this context, the development of safe brain-centered drug-free treatments for children and adolescents with ADHD merits undoubtedly further investigation.

For example, transcranial direct current stimulation (tDCS) directly and non-invasively modulates cortical excitability and, consequently, associated behavior and cognitive functions via a weak electrical current (generally between 0.5 and 2 mA) delivered through one or more electrodes placed on the scalp (Jamil et al., 2017). Specifically, the current modulates spontaneous discharge rates and generates subthreshold polarity-dependent shifts in resting membrane potentials, increasing (anode electrode) or decreasing (cathode electrode) the excitability of underlying neurons and leading to respective increases or decreases in cortical function and synaptic strength (Jamil et al., 2017). In this way, tDCS significantly enhances neuroplasticity since its neurophysiological effects last for over an hour (Nitsche and Paulus, 2000; Kuo et al., 2016), especially when combined with a training or cognitive task (Fritsch et al., 2010).

The application of tDCS in pediatric population is an emerging area of intensive research, supported by promising results in a wide range of neurodevelopmental disorders (Costanzo et al., 2016; Finisguerra et al., 2019; Lazzaro et al., 2021; Battisti et al., 2022). Over the past decades, studies exploring the effects of tDCS in children and adolescents with ADHD have increased significantly. Most studies have examined the effects of tDCS on ADHD-related executive function deficits (inhibitory control, WM, attention, cognitive flexibility) and reward processing (Salehinejad et al., 2022). However, results are still sparse and mixed. Some studies documented null effects of tDCS (Westwood et al., 2021b; Salehinejad et al., 2022), whereas other studies reported an improvement of inhibitory control and WM after anodal tDCS over the left DLPFC (Munz et al., 2015; Soltaninejad et al., 2019; Nejati et al., 2020; Berger et al., 2021; Klomjai and Aneksan, 2022). In line with this evidence, studies applying tDCS in adults without ADHD promote left DLPFC as one of the key target area for modulating executive functions, especially inhibitory control (Friehs et al., 2021) and WM (Brunoni et al., 2014; Hill et al., 2016; Mancuso et al., 2016).

Further studies are still needed to understand the actual potential of this technique in the treatment of ADHD. In addition, direct comparison between tDCS and MPH could provide interesting insights into the magnitude of the effect on cognitive function of brain stimulation compared with MPH treatment.

The aim of the present study is to test whether tDCS can induce improvement in inhibitory control and WM of children and adolescents with ADHD and to explore whether the effect of a single session of tDCS can be comparable to that obtained after a single dose of MPH.

## 2. Materials and methods

### 2.1. Participants

All participants received a comprehensive neuropsychiatric assessment at the Child and Adolescent Neuropsychiatry Unit of the Bambino Gesù Children’s Hospital (Rome). Neuropsychiatrists and development psychologists conducted clinical eligibility screenings, evaluating cognitive level, ADHD symptoms and the occurrence of comorbid psychiatric disorders.

Cognitive level was assessed using the Wechsler Intelligence Scale for Children Fourth Edition (perceptual reasoning index) (Orsini et al., 2012) or the Raven Progressive Matrices (Raven and Court, 1998).

The diagnosis of ADHD and psychiatric comorbidities was based on the developmental history, a thorough clinical examination, the semi-structured interview Kiddie Schedule for Affective Disorders and Schizophrenia Present and Lifetime Version for DSM-5 (K-SADS-PL DSM-5) (Kaufman, 2019) and parent-report questionnaires (CBCL, Child Behavior Checklist; CPRS, Conners’ Parent Rating Scales; SNAP-IV; C-GAS, Children Global Assessment Scale) (Swanson et al., 1981; Shaffer, 1983; Achenbach, 1991; Conners et al., 1998).

Children and adolescents with the following characteristics were considered for the inclusion in the study:

1) diagnosis of ADHD (combined presentation) according to the DSM-5 criteria (1);
2) Intelligence Quotient (IQ): higher than or equal to 85 (IQ ≥ 85);
3) age between 8 years to 13 years and 11 months;
4) normal or corrected-to-normal vision;
5) being drug naïve and needing drug treatment for ADHD symptoms.

Patients were excluded if they presented:

6) autism spectrum disorders or specific psychiatric disorders (e.g., bipolar disorders, schizophrenia spectrum disorders, or adjustment disorder) as comorbid conditions;
7) a history of neurological (e.g., seizures, migraines, injury resulting in a loss of consciousness) or medical (e.g., scalp or skin condition such as psoriasis or eczema, the presence of any metallic implants including intracranial electrodes, surgical clips, shrapnel or a pacemaker) or genetic conditions;
8) a pre-existing medical condition (e.g., heart, kidney, or liver diseases) contraindicated for the administration of MPH.

After clinical eligibility screening, 26 children and adolescents (24 males and 2 females, age: 10.63 ± 1.41 years, IQ: 106.42 ± 10.85) with combined presentation of ADHD were included in the study. All participants and their parents were fully instructed about the procedures and the purpose of the study, and both parents and adolescents aged 12 years and older provided written consent before entering the study.

Ethical approval for the study was granted by the local research ethics committee (process number 2185_OPBG_2020) and was registered at ClinicalTrials.gov (ID: NCT04964427) on the 13^th^ of July 2021. The rationale and design of this trial was discussed in a published Study Protocol (D’Aiello et al., 2022a). The study was performed following the Declaration of Helsinki.

### 2.2. Study design

A randomized, single-blind, within-subjects, and sham-controlled design was conducted.

**Figure 1:**
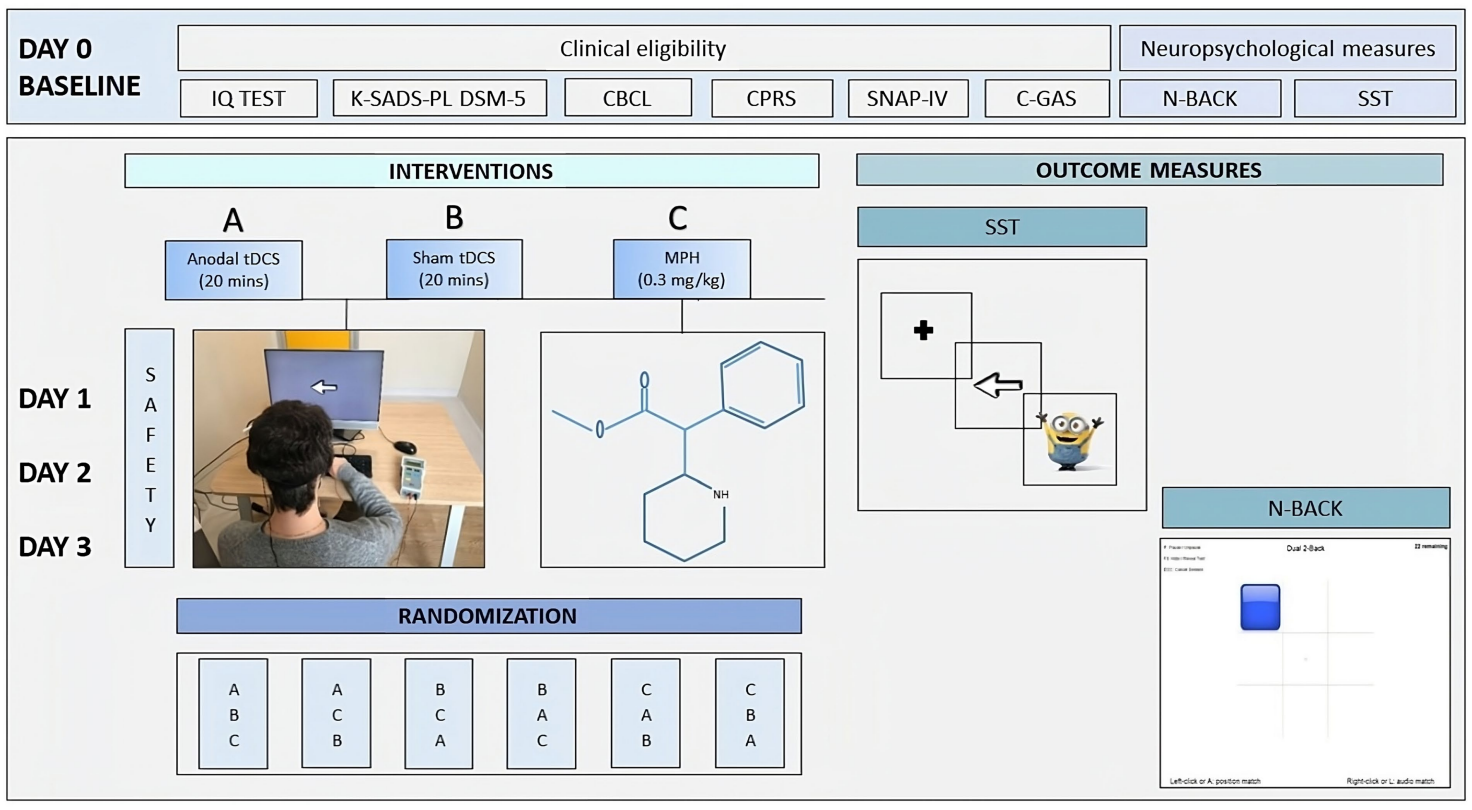
Overview of the study design. DAY 0, Baseline; DAY 1, DAY 2, DAY 3, Day of conditions administration; **(A)** single session of anodal tDCS; **(B)** single session of sham tDCS; **(C)** single dose of MPH (Ritalin®); CBCL, Child Behavior Checklist; CPRS, Conners’ Parent Rating Scales; SNAP-IV; K-SADS-PL DSM-5, Kiddie Schedule for Affective Disorders and Schizophrenia Present and Lifetime Version for DSM-5; C-GAS, Children Global Assessment Scale; ABAS-II, Adaptive Behavior Assessment System; N-Back; SST, Stop Signal Task; Safety and Tolerability Questionnaire.

After clinical eligibility screening at baseline (Day 0), participants were exposed to three conditions with a 24-h interval-sessions (Day 1, Day 2, Day 3, see Figure 1A) a single session of active anodal tDCS; Figure 1B a single session of sham tDCS; and Figure 1C a single dose of MPH (Ritalin®) administered according to the National Institute for Clinical Excellence (NICE) guidelines for ADHD (Atkinson and Hollis, 2010). After recruitment, participants were assigned to one of six possible sequences: ABC (4 participants), ACB (4 participants), BAC (5 participants), BCA (4 participants), CBA (5 participants), or CAB (5 participants). Assignment to one of six possible combinations was according to a computer-generated randomization order. Randomization information was stored by an independent researcher until the data collection was completed.

The participants and their families were blinded to the type of tDCS sessions (sham/active). Outcome measures were recorded at Day 0, Day 1, Day 2, and Day 3.

### 2.3. Interventions

#### 2.3.1. Transcranial direct current stimulation

Direct current was delivered by a battery-operated direct current stimulator (Brain-Stim stimulation by E.M.S. S.R.L-Bologna, Italy) through a pair of identical square (25 cm^2^) saline sponge electrodes held in place by elastic bands. The anodal electrode was placed over the left DLPFC, in accordance with the International System 10–20, on the sites corresponding to F3, while the cathodal electrode was located over the contralateral supraorbital area (OFC), corresponding to Fp2.

During the anodal tDCS condition, the current slowly increased during the first 30 s to 1 mA (ramp-up) and, at the end of stimulation, the current slowly decreased to 0 mA during the last 30 s (ramp-down). Based on previous studies conducted on children and adolescents with neurodevelopmental disorders (Minhas et al., 2012; Kessler et al., 2013; Costanzo et al., 2016; Lazzaro et al., 2021), a current intensity of 1 mA was applied, reduced from what is generally administered to adults, to take into account the characteristics of the pediatric population, such as smaller head size, thinner scalp, and less cerebrospinal fluid than adults, which would influence current distribution and density at the site of stimulation (Minhas et al., 2012; Kessler et al., 2013; Opitz et al., 2015).

Between ramp-up and ramp-down, constant current was delivered for 20 min, with a density of 0.04 mA/cm^2^.

During the sham tDCS condition, stimulation was delivered the same positioning as active tDCS, respectively left anodal on DLPFC and right electrode reference on OFC. The stimulation intensity was set to 1 mA, but the current was applied for 30 s and was reduced without participant awareness. This placebo condition induces sensations (e.g., tingling) associated with tDCS and, is therefore indistinguishable by participants from the active condition (Gandiga et al., 2006).

tDCS set-up was based on previous studies in children and adolescents with ADHD (Salehinejad et al., 2020b) demonstrating that anodal but not cathodal DLPFC significantly improves inhibitory control and WM in participants with ADHD (Salehinejad et al., 2019). Furthermore, the left anodal DLPFC/right cathodal OFC (reference electrode) montage was proved to be the most effective electrode placement (Salehinejad et al., 2019).

#### 2.3.2. Methylphenidate

Depending on the age and weight of the child, a single dose of 5–10 mg of immediate-release MPH (Ritalin®) was administered by a neuropsychiatrist in accordance with NICE and AIFA (Agenzia Italiana del Farmaco) guidelines for the treatment of ADHD. Neuropsychiatric preventive and naturalistic assessment (anamnestic history, the mental state examination, and the neurological examination) according to the AIFA guidelines for ADHD was conducted by a neuropsychiatrist before recruitment. At that time, cardiovascular risk factors associated with MPH assumption (e.g., Brugada syndrome) were excluded by clinicians. After this first evaluation, the participants underwent medical examinations. Specifically, the electrocardiogram and the correction of the QT segment were preventively evaluated by a cardiologist. Moreover, blood exams were carried out to exclude any other medical condition associated with ADHD and that may mime this disorder’s symptoms (e.g., thyroiditis).

### 2.4. Outcome measures

**Figure 2:**
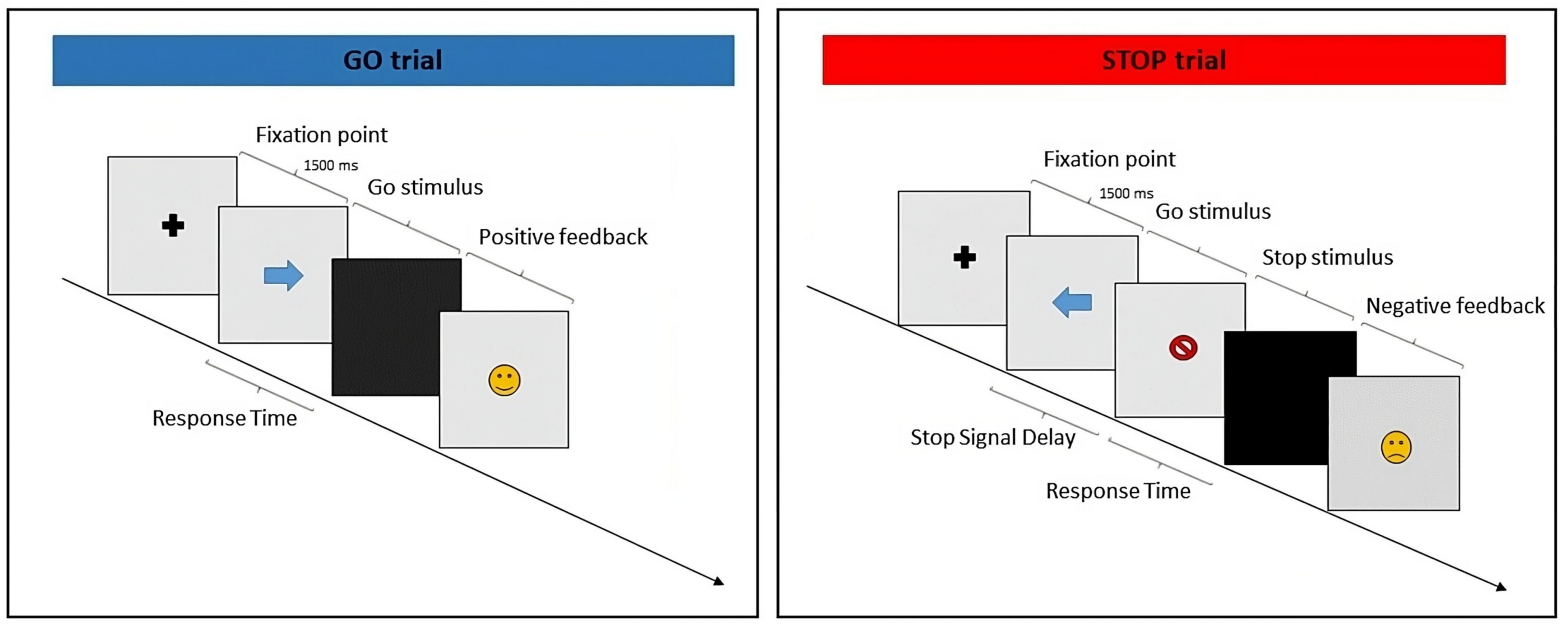
Depiction of the sequence of events in a Stop Signal Task.

The primary outcome measure was response inhibition (Stop Signal Reaction Time—SSRT, see Figure 2) measured with the Stop Signal Task – SST (Logan and Cowan, 1984). The SST is a go no-go task that assesses the ability to suppress a dominant response. The SST was performed with PsychoPy® software (Open Science Tools Ltd., Nottingham, United Kingdom) (Peirce et al., 2019), and was designed in accordance with the SST consensus guide (Verbruggen et al., 2019). It consisted of randomly intermixed go and stop trials (75 and 25%, respectively). All trials began with the presentation of a cross in the center of the computer monitor. After 1,000 ms, a stimulus target (go signal) replaced the cross. In the go trials, children were instructed to press the space bar as fast as possible after the go signal appeared. The go signal persisted on the screen up to a maximum of 1,500 ms. In the stop trials, after a variable delay, a stop stimulus target appeared following the go signal (Stop-Signal Delay, SSD). Children were required to abstain from responding. The duration of SSD was monitored with a simple staircase procedure (50 ms step) to maintain the probability of inhibition around 50% of the trials. SSD was increased or decreased by a single step after a successful or failed arrest. When the participant repeatedly failed to inhibit the response, the SSD could reach a minimum delay of 0 ms, thus making the stop signal a no-go signal (a no-go signal can be considered a stop signal with SSD = 0); when the participant repeatedly succeeded in inhibiting the response, the SSD could increase to a maximum of the entire duration of the target stimulus presentation (1,500 ms). However, among subjects, the maximum value reached was 814.79 ms, while the average of the max SSD across participants was 412.95 ± 123.85. SSRT was calculated (in ms) by subtracting a mean estimate of SSD from the observed mean reaction times (RTs) in no-stop trials. The duration of the task was about 14 min.

Because intra-individual variability in RTs is often observed in ADHD (Castellanos et al., 2005, 2006; Fassbender et al., 2009; Tamm et al., 2012), an additional analysis was performed on the variability of reaction times (VRTs) in correct go trials to see if different interventions reduced performance fluctuations.

The outputs of the SST consisted of the following parameters: Accuracy (percentage of corrected trials, including go and no-go trials), SSRT, RTs, VRTs.

In Figure 2, participants respond to the direction of arrows by pressing the corresponding arrow key in the go task. In one of the trials, the arrow is replaced by a stop symbol after a variable SSD. Each response is followed by positive or negative feedback.

The N-Back task is one of the most frequently used culture-free instruments to assess WM. The visual–spatial condition consists of presenting a series of visual stimuli (blue boxes) at a given position on the screen. After a training phase, participants had to indicate whether the position of each box presented was the same as that presented in previous trials. For example, in 2-Back task, participants had to indicate whether the current position was the same as the position in trial *n* − 2. When the accuracy was higher than 80%, the difficulty of the N-Back task increased (e.g., going from 1-Back to 2-Back). The N-Back index was determined based on the last unachieved span (i.e., when the accuracy percentage < 80%). For example, if the participant reached the 1-Back span (accuracy percentage ≥ 80%) and achieved only 30% accuracy in the 2-Back span, the N-Back index would be 2.3. The task took about 2 min to complete.

Outcome measures were collected at Day 0 and 10 min after the start of stimulation (Martin et al., 2014) or 90 min after MPH administration (maximum peak for MPH effects as mentioned in Ritalin® label), when each treatment achieved its maximum effect.

N-back and SST tasks were administered during tDCS, in agreement with studies (Miniussi and Vallar, 2011; Martin et al., 2014; Dedoncker et al., 2016) that have shown that accuracy in online tasks seems to be more effective than in offline tasks.

The order of N-back and SST was counterbalanced across sessions.

Overall, the evaluation took about 20 min to complete.

#### 2.4.1. Safety and tolerability of tDCS

Safety and tolerability are important dimensions for validating and translating a treatment in the daily clinical routine. Therefore, symptoms and side effects were evaluated by using a standard questionnaire (Brunoni et al., 2011) completed by each participant after each tDCS session. The questionnaire assesses adverse effects such as headache, neck pain, scalp pain, tingling, itching, burning sensation, skin redness, sleepiness, trouble concentrating, and acute mood change. Participants quantified the intensity of the symptoms or side effects related to tDCS (1—absent; 2—mild; 3—moderate; 4—severe).

### 2.5. Simple size calculation

Sample size was calculated using *a priori* analysis in G*Power, version 3.1.9.7 (Faul et al., 2007). As previously reported in the study protocol (D’Aiello et al., 2022a), the sample size of 24 was calculated using a repeated-measures Analysis of Variance (ANOVA) model with four within-factors (baseline, anodal tDCS, sham tDCS, and MPH), considering an estimated *f* = 0.25, α value = 0.05 (i.e., probability of false positives of 5%), and β = 0.80 (i.e., at least 80% power).

### 2.6. Statistical analyses

Repeated-measures ANOVAs were conducted separately on SSRT, VRTs and Accuracy of SST with Condition (Day 0, anodal tDCS, sham tDCS, single dose of MPH) as a within-subjects factor. *Post-hoc* comparisons were run using Tukey’s honest significance test. Partial eta square (η_p_^2^) was used as a measure of effect size.

The Friedman’s ANOVAs were employed to conduct nonparametric tests on the RTs of the SST and the visual–spatial N-Back index, as they did not follow a normal distribution. *Post-hoc* analyses were conducted by using Wilcoxon signed-rank tests to check for potential differences among conditions (Day 0, anodal tDCS, sham tDCS, and MPH). Cohens’ *d* was used as a measure of effect size.

To exclude sequences effect, non-parametric analysis was run for each outcome measure (see Supplementary materials: 1. Sequence effect analysis and 2. Sequence effect result).

A *p* value ≤0.05 was considered statistically significant.

## 3. Results

**Figure 3:**
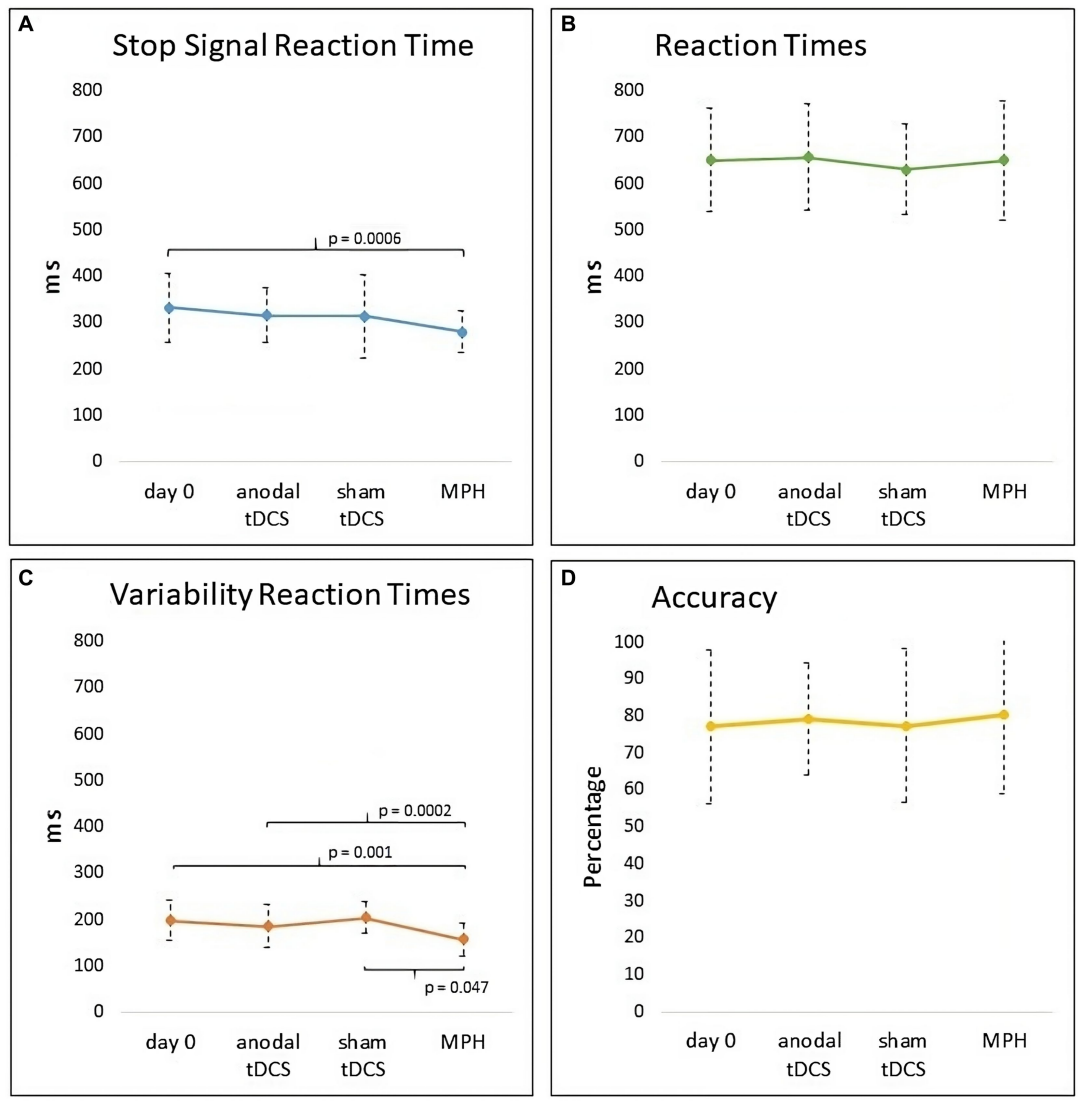
Graph of outcome measures (SSRT, RTs, VRTs, Accuracy) at Day 0 and in anodal tDCS, sham tDCS and MPH conditions. **(A)** A significant reduction of SSRT was found after a single dose of MPH compared with Day 0. **(B)** No difference was found in RTs. **(C)** A significant reduction of VRTs after a single dose of MPH compared with Day 0, anodal tDCS, and sham tDCS. **(D)** No difference was found in the percentage of Accuracy of SST.

Repeated-measures ANOVA documented a significant Condition Effect (*F*_3,75_ = 3.92, *p* = 0.011, η_p_^2^ = 0.13) in the primary outcome, the SSRT (see Figure 3 panel A). *Post-hoc* comparisons showed a significant reduction of SSRT after a single dose of MPH compared with Day 0 (*p* = 0.006). However, no significant differences were found between MPH and anodal tDCS (*p* = 0.11) or between MPH and sham tDCS (*p* = 0.15).

No further significant differences were found (anodal tDCS *vs* Day 0: *p* = 0.71; anodal tDCS *vs* sham tDCS: *p* = 0.99; sham tDCS *vs* Day 0: *p* = 0.62).

When considering RTs, Friedman’s ANOVA did not reveal significant differences (*χ*^2^_(3)_ = 0.88, *p* = 0.83) among the conditions (see Figure 3 panel B).

Repeated-measures ANOVA showed a significant Condition effect (F_3,75_ = 7.90, *p* = 0.001, η_p_^2^ = 0.24) in VRTs (see Figure 3 panel C). *Post-hoc* documented a significant reduction of VRTs after a single dose of MPH compared with Day 0 (*p* = 0.001), anodal tDCS (*p* = 0.0002), and sham tDCS (*p* = 0.047). No further significant differences were found (anodal tDCS *vs* Day 0: *p* = 0.92; anodal tDCS *vs* sham tDCS: *p* = 0.26; sham tDCS *vs* Day 0: *p* = 0.61).

Repeated-measures ANOVA showed no significant Condition effect (F_3, 75_ = 0.14, *p* = 0.93) in the Accuracy of SST (see Figure 3 panel D).

**Figure 4:**
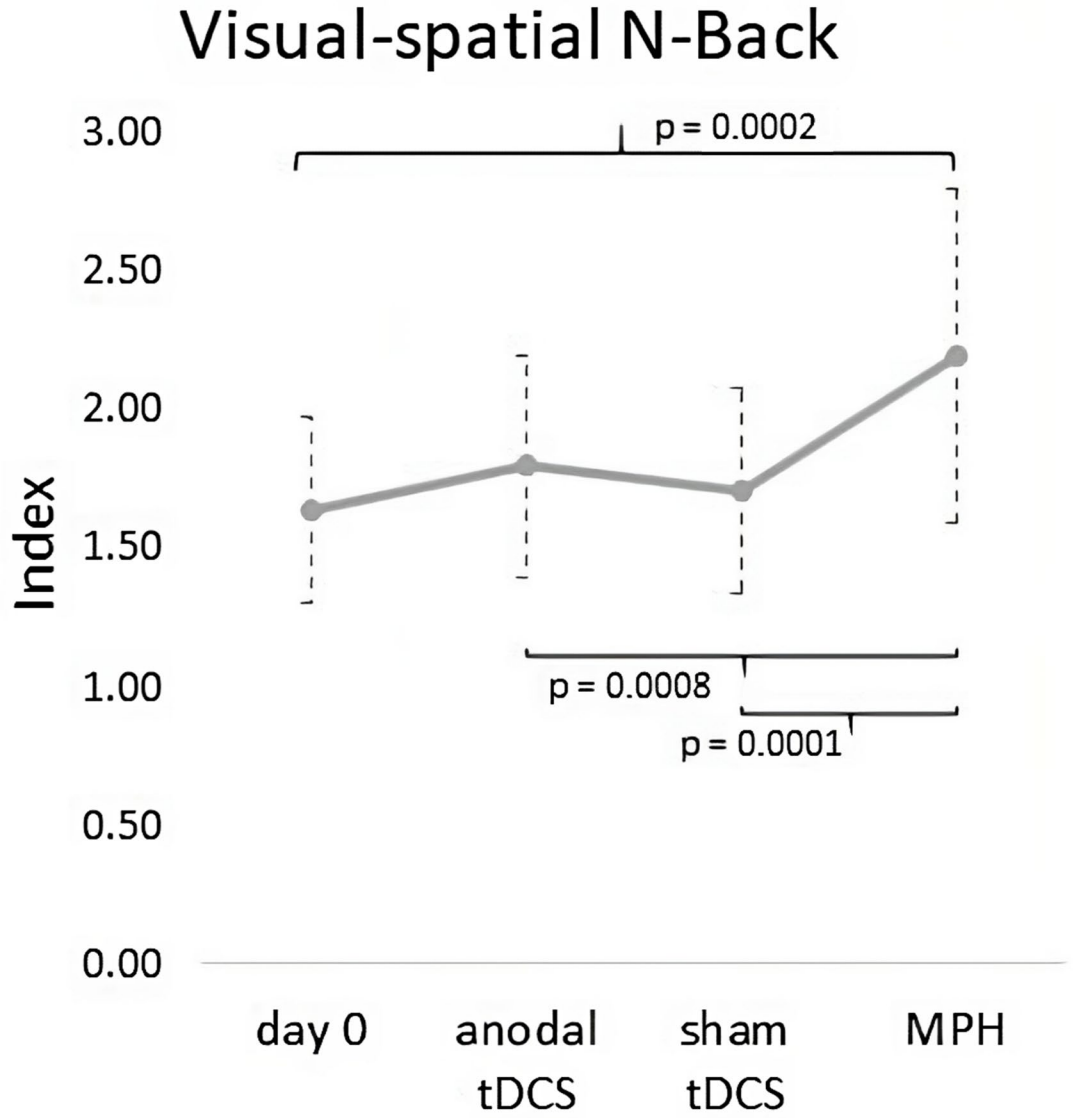
Graph of outcome measures (Visual–spatial N-Back) at Day 0 and in anodal tDCS, sham tDCS and MPH conditions. A significant increment of visual–spatial N-Back index was found after a single dose of MPH compared with Day 0, anodal tDCS and sham tDCS.

Friedman’s ANOVA revealed a significant difference among the conditions (*χ*^2^_(3)_ = 17.75, *p* = 0.0005). Wilcoxon signed-rank tests documented a significant increment of visual–spatial N-Back index after a single dose of MPH compared with Day 0 (*Z* = 3.88, *p* < 0.0001, Cohens’ *d* = 2.3), anodal tDCS (*Z* = 2.62, *p* = 0.008, Cohens’ *d* = 1.2) and sham tDCS (*Z* = 3.06, *p* = 0.0002, Cohens’ *d* = 1.5). No further significant differences were found (anodal tDCS *vs* Day 0: *p* = 0.23; anodal tDCS *vs* sham tDCS: *p* = 0.57; sham tDCS *vs* Day 0: *p* = 0.22) (see Figure 4).

Supplementary Table S4 depicts mean and SD of outcome measures (SSRT, RTs, VRTs, Accuracy, Visual–spatial N-Back index) for Day 0, anodal tDCS, sham tDCS and MPH conditions (see Supplementary materials).

### 3.1. Safety measures

Regarding the safety questionnaire used to assess post-stimulation adverse effects (Miniussi and Vallar, 2011), most participants (24/26) did not experience any adverse effects other than itching during both active and sham stimulation. Only two participants with a very fair skin complexion experienced redness after the active stimulation session.

## 4. Discussion

The present study is the first attempt to compare the effects of tDCS with those of established pharmacological treatment.

In this randomized, single-blind, sham-controlled study, children and adolescents with ADHD were exposed to a single session of anodal tDCS, sham tDCS, and a single dose of MPH.

After a single dose of MPH, results showed a significant improvement in inhibitory control (in terms of VRTs and SSRT) and visual–spatial WM compared with baseline, one session of anodal tDCS and sham tDCS. These findings corroborate previous evidence, showing MPH beneficial effects on inhibitory control and visual–spatial WM (Bedard et al., 2004; Spencer et al., 2009; Campez et al., 2022; D’Aiello et al., 2022b; Vertessen et al., 2022).

However, several explanations could be proposed for the lack of results on cognitive variables examined after left anodal tDCS on DLPFC. First, a possible reason may lay into the stimulated brain region – i.e., DLPFC. The choice of stimulating this brain region was based on previous studies demonstrating that the DLPFC abnormalities are physiologically associated with the cognitive deficits of ADHD (Faraone et al., 2015). For these reasons, the left DLPFC was widely targeted via tDCS to improve both inhibitory control and WM in ADHD (Salehinejad et al., 2019, 2020b; Breitling et al., 2020). During inhibitory tasks, such as SST, hypo-activation of the DLPFC has been observed in ADHD (Inoue et al., 2012; Monden et al., 2015) and the bilateral hypoactivity of the DLPFC (Lei et al., 2015) at the resting state (Hoekzema et al., 2014) and during cognitive tasks (Langleben et al., 2001). However, it should be considered that inhibitory control and visual–spatial WM involve a widespread network of fronto-parietal areas (Hart et al., 2012), the cerebellum, as documented by functional neuroimaging and tDCS studies in individuals without ADHD (Mannarelli et al., 2020) as well as the temporo-parietal regions (Garavan et al., 1999; Pliszka et al., 2006; Rubia et al., 2008, 2011; Sripada et al., 2014; Mencarelli et al., 2022). These brain regions have been little targeted (Westwood et al., 2021b) and should, therefore, be further explored in tDCS studies with children and adolescents with ADHD. Indeed, a recent review (Westwood et al., 2021b) underlined that out of 17 tDCS studies conducted in children and adolescents with ADHD, the majority have stimulated DLPFC (10/17), only one study targeted the ventromedial prefrontal cortex (Nejati et al., 2020), another one applied tDCS to the posterior parietal cortex (Salehinejad et al., 2020a), and the last delivered anodal tDCS to the right inferior frontal gyrus (Breitling et al., 2016, 2020; Breitling-Ziegler et al., 2021; Westwood et al., 2021a, 2022).

Second, a potential explanation could be due to the specificity of the neurophysiological actions associated with tDCS. Indeed, it is well-known that tDCS can induce the alteration of cortical excitability that depends on the dendro-axonic orientation of the pyramidal cells (Das et al., 2016; Yavari et al., 2018). However, once passing via the scalp, current flow through the brain is diffuse, reaching both the brain regions covered by the electrodes with a peak of intensity as well as large swaths of cortical and subcortical structures, although with lower intensity (Kronberg et al., 2017). Whereas, compared to tDCS, MPH has a stronger and direct modulatory effect on subcortical structures such as the striatum and putamen (Rubia et al., 2011) – which are mostly associated with inhibitory control and visual–spatial WM (Haber, 2017).

Third, a single stimulation of the anodal tDCS may not have been sufficient to give an effect. Although a single dose of MPH has been shown to affect brain network connectivity and cognitive symptoms (Epstein et al., 2007; Rubia et al., 2009, 2011; Kaiser et al., 2022), evidence converges that non-invasive brain stimulation techniques achieve their maximum effectiveness when the stimulation sessions are repeated (Fecteau et al., 2014; Meron et al., 2015; Lefaucheur et al., 2017). Multiple sessions of tDCS induce cumulative neurobiological effects over time, generating more robust neuroplasticity processes (Nitsche and Paulus, 2000; Boggio et al., 2009; Monte-Silva et al., 2013) that have greater efficacy on behavioral symptoms and neurocognitive measures.

The current results have helped confirm the safety and feasibility of tDCS in the pediatric population (Fritsch et al., 2010; Salehinejad et al., 2019, 2020b) as side effects are negligible, with at most scalp redness.

Finally, online administration of tDCS may have influenced the results. Although tDCS during task performance has been shown to increase the synaptic strength of neural networks already activated by concurrent tasks (Miniussi and Vallar, 2011; Martin et al., 2014; Dedoncker et al., 2016), some studies have documented that behaviors are more influenced by offline than online tDCS (Stagg et al., 2011; Amadi et al., 2015; Grasso et al., 2020, 2021).

Our study has some limitations. The first refers to the absence of a placebo pharmacological-control intervention to compare the effects obtained after MPH administration. Future studies involving four conditions (MPH, pharmacological placebo, anodal tDCS, and sham tDCS) could be useful to clarify the contribution of a single dose of MPH compared with placebo and the non-invasive brain stimulation conditions in the same group of children with ADHD.

The number of participants can also be considered a limitation of the present study. To confirm the superiority of the effects of MPH over tDCS, it will be necessary to collect data from larger groups.

In addition, neurophysiological and neuroimaging studies, such as EEG and/or MRI studies, could help explain the reduced effect of a single non-invasive brain stimulation session.

In conclusion, our protocol involving a single tDCS session in ADHD did not demonstrate consistent improvements in neuropsychological measures. Different protocols could be developed that provide significant effects. Different montages should be implemented to target other potential brain regions involved in inhibitory control and visual–spatial WM (i.e., posterior parietal cortex) beyond DLPFC. Other transcranial electrical stimulation techniques (e.g., high-definition tDCS) could be used so as to reach more deeply and focally the brain structures involved in ADHD symptoms. In addition, multi-session tDCS protocols could make more significant changes than a single session for ADHD, possibly in combination with usual treatments such as cognitive-behavioral psychotherapy and medication. However, our study has the merit of directly comparing tDCS with a pharmacological intervention (D’Aiello et al., 2022a), whereas most studies have examined the effects of tDCS as an adjunct to pharmacological treatment (Brunoni et al., 2013; Bennabi and Haffen, 2018; McLaren et al., 2018; Rigi Kooteh et al., 2019; Wang et al., 2021a). Direct comparison between tDCS and pharmacological intervention is important because it allows comparison of the parameters of tolerability, safety, and feasibility, which are crucial aspects of translating scientific findings into actual clinical practice.

In light of previous considerations, it should not be therefore excluded that tDCS could represent a promising treatment for ADHD-associated executive dysfunctions, and it is still an open area of research which merits to be further investigated.

## Data availability statement

The original contributions presented in the study are included in the article/Supplementary material, further inquiries can be directed to the corresponding author.

## Ethics statement

The studies involving human participants were reviewed and approved by the Ethics Committee of the Bambino Gesù Children Hospital (2185_OPBG_2020). Written informed consent to participate in this study was provided by the participants’ legal guardian/next of kin.

## Author contributions

BD, DM, and SV: conceptualization. BD, DM, GL, PP, and SV: methodology. SV and DM: supervision. BD, GL, and AB: writing original draft. BD, DM, GL, AB, PDR, SDV, IP, FC, and SV: writing review and editing. All authors have read and agreed to the published version of the manuscript.

## Funding

This work was supported by the Italian Ministry of Health with “Current Research” funds.

## Conflict of interest

The authors declare that the research was conducted in the absence of any commercial or financial relationships that could be construed as a potential conflict of interest.

## Publisher’s note

All claims expressed in this article are solely those of the authors and do not necessarily represent those of their affiliated organizations, or those of the publisher, the editors and the reviewers. Any product that may be evaluated in this article, or claim that may be made by its manufacturer, is not guaranteed or endorsed by the publisher.
