# Supplementary material for: Methylphenidate is more effective to improve inhibitory control and working memory compared to tDCS in children and adolescents with attention deficit/hyperactivity disorder: a proof-of-concept study
Source: Front Neurosci. 2023 Jul 7;17:1170090. doi: 10.3389/fnins.2023.1170090 (PMC10360130; doi:10.3389/fnins.2023.1170090)
Supplement: Supplementary file 1 [file Data_Sheet_1.docx]

**Supplementary Materials**

**1. Sequence effect analysis**

The Kruskal-Wallis ANOVAs were conducted on age, IQ, SSRT, RTs, VRTs, Accuracy and the Visual-spatial N-Back index and Sequences as grouping variable.

Bonferroni’s correction for multiple comparisons was applied to account for the comparisons of the six Sequences. A p value ≤ 0.05 was considered statistically significant.

**2. Sequence effect results**

The six Sequences did not differ for age [H (5, N = 26) = 1.95, p = 0.85] or IQ [H (5, N = 26) = 5.91, p = 0.31].

No significant difference between Sequences was found in SSRT at T0 [H (5, N = 26) = 2.05, p = 0.84], in sham tDCS [H (5, N = 26) = 1.62, p = 0.89] or after active tDCS [H (5, N = 26) = 3.16, p = 0.67]. A significant Sequences effect was found in SSRT in MPH [H (5, N= 26) =12.81, p = 0.025]. Sequence ABC differs (p = 0.012) significantly from sequence BAC (see Table S1).

Table S1. Mean (SD) of SSRT at Day 0 and in anodal tDCS, sham tDCS and MPH conditions for Sequences.

| SSRT | | | | |
| --- | --- | --- | --- | --- |
| Sequences | T0 | Sham  tDCS | Active tDCS | MPH |
| ABC | 332.40 (74.73) | 313.28 (89.64) | 315.49 (59.44) | 279.51 (44.91) |
| BAC | 362.76 (29.73) | 315.93 (85.17) | 370.23 (76.24) | 323.67 (32.54) |
| CAB | 307.85 (104.19) | 270.41 (49.77) | 298.52 (42.56) | 232.30 (16.82) |
| BCA | 332.57 (28.04) | 319.75 (143.73) | 300.92 (55.33) | 267.77 (56.18) |
| CBA | 320.34 (95.68) | 317.11 (69.61) | 311.01 (34.27) | 292.83 (28.43) |
| ACB | 340.13 (110.52) | 348.31 (126.88) | 306.02 (59.37) | 274.53 (37.77) |
| ACB | 334.96 (49.12) | 310.12 (65.04) | 312.86 (85.67) | 299.01 (47.12) |

SSRT = Stop Signal Reaction Time (in ms).

No significant difference between Sequences was found in RTs at T0 [H (5, N = 26) = 6.29, p = 0.27], in sham tDCS [H (5, N = 26) = 5.01, p = 0.41], active tDCS [H (5, N= 26) = 7.48, p = 0.18] or MPH [H (5, N= 26) = 9.47, p = 0.09].

No significant difference between Sequences was found in VRTs at T0 [H (5, N = 26) =10.52, p = 0.062], in sham tDCS [H (5, N= 26) = 3.02, p = 0.69], active tDCS [H (5, N= 26) = 6.5, p = 0.25] or MPH [H (5, N = 26) = 8.31, p = 0.13] (see Table S2).

Table S2. Mean (SD) of RTs and VRTs at Day 0 and in anodal tDCS, sham tDCS and MPH conditions for Sequences.

|  | RTs | | | | VRTs | | | |
| --- | --- | --- | --- | --- | --- | --- | --- | --- |
| Sequences | T0 | Sham  tDCS | Active tDCS | MPH | T0 | Sham tDCS | Active tDCS | MPH |
| ABC | 647.83 (57.92) | 646.11 (82.71) | 759.04 (150.72) | 709.10 (93.62) | 182.50 (22.17) | 217.50 (42.72) | 230.00 (35.59) | 162.50 (35.93) |
| BAC | 681.85 (79.71) | 578.54 (64.41) | 578.22 (46.00) | 520.91 (43.49) | 232.00 (43.81) | 178.00 (32.71) | 186.00 (37.14) | 132.00 (19.23) |
| CAB | 619.18 (75.44) | 679.16 (165.12) | 727.12 (101.58) | 634.46 (47.52) | 182.50 (20.61) | 192.50 (47.87) | 205.00 (23.80) | 135.00 (23.80) |
| BCA | 606.05 (133.29) | 576.05 (100.75) | 614.73 (156.11) | 651.67 (151.90) | 187.50 (45.73) | 170.00 (42.42) | 185.00 (33.16) | 170.00 (42.42) |
| CBA | 743.11 (165.22) | 662.50 (82.87) | 699.92 (150.53) | 743.89 (196.50) | 227.40 (38.97) | 167.54 (57.25) | 222.40 (23.63) | 167.00 (50.19) |
| ACB | 569.85 (53.60) | 641.62 (80.71) | 570.84 (87.75) | 649.60 (59.43) | 157.50 (40.31) | 190.00 (63.24) | 195.00 (38.72) | 182.50 (15.00) |

RTs = Reaction Times (in ms); VRTs = Variability of Reaction Times (in ms)

No significant difference between Sequences was found in Accuracy at T0 [H (5, N = 26) = 1.10 p = 0.95], in sham tDCS [H (5, N = 26) = 1.94, p = 0.85], active tDCS [H (5, N = 26) = 7.09, p = 0.21] or MPH [H (5, N = 26) = 5.83, p = 0.32].

No significant difference between Sequences was found in N-back index at T0 [H (5, N = 26) = 2.69, p = 0.75], sham tDCS [H (5, N = 26) = 1.3, p =0.93], or MPH [H (5, N =25) = 5.55, p = 0.35]. A significant Sequences effect was found in active tDCS [H (5, N = 26) =12.08, p = 0.03]. However, no multiple comparisons were found (see Table S3).

Table S3. Mean (SD) of Visual-spatial N-Back index and Accuracy at Day 0 and in anodal tDCS, sham tDCS and MPH conditions for Sequences.

|  | Accuracy | | | | Visual-spatial N-back index | | | |
| --- | --- | --- | --- | --- | --- | --- | --- | --- |
| Sequences | T0 | Sham tDCS | Active tDCS | MPH | T0 | Sham tDCS | Active tDCS | MPH |
| ABC | 81.75 (3.94) | 82.25 (4.32) | 80.00 (3.46) | 87.50 (0.57) | 1.87 (0.6) | 1.87 (0.51) | 1.63 (0.13) | 2.58 (0.69) |
| BAC | 83.00 (3. 16) | 84.00 (3.63) | 84.80 (2.03) | 86.60 (1.14) | 1.65 (0.49) | 1.61 (0.27) | 1.92 (0.39) | 2.47 (0.80) |
| CAB | 65.75 (3.60) | 84.00 (4.08) | 84.25 (2.75) | 86.00 (2.16) | 1.61 (0.12) | 1.71 (0.37) | 1.57 (0.16) | 1.78 (0.44) |
| BCA | 80.75 (4.71) | 82.75 (2.87) | 83.25 (2.21) | 86.25 (2.87) | 1.56 (0.18) | 1.75 (0.44) | 2.49 (0.22) | 2.11 (0.56) |
| CBA | 83.80 (5.93) | 84.70 (2.98) | 83.80 (1.61) | 85.16 (1.62) | 1.47 (0.15) | 1.62 (0.15) | 1.65 (0.28) | 2.11 (0.50) |
| ACB | 84.50 (2.68) | 83.00 (5.41) | 76.50 (7.85) | 88.00 (2.37) | 1.67 (0.11) | 1.77 (0.59) | 1.53 (0.23) | 2.15 (0.49) |

Accuracy = percentage of correct responses

**3. Supplementary results**

Table S4. Mean (SD) of outcome measures (SSRT, RTs, VRTs, Accuracy, Visual-spatial N-Back index) at Day 0 and for anodal tDCS, sham tDCS and MPH conditions.

| Outcome Measures | Conditions | | | |
| --- | --- | --- | --- | --- |
|  | Day 0 | anodal tDCS | sham tDCS | MPH |
|  | M  (SD) | M  (SD) | M  (SD) | M  (SD) |
| SSRT | 332.4  (74.73) | 315.5  (59.45) | 313.3  (89.64) | 279.5^  (44.9) |
| RTs | 649.87  (111.24) | 656.83  (113.54) | 629.89  (97.53) | 650.13  (129.19) |
| VRTs | 197.6  (43.21) | 203.9  (33.96) | 184.9  (46.63) | 157.5**  (35.87) |
| Accuracy | 77.11  (20.70) | 79.19  (15.16) | 77.36  (20.68) | 80.22  (21.32) |
| Visual-spatial  N-Back index | 1.64  (0.34) | 1.80  (0.40) | 1.71  (0.37) | 2.2**  (0.60) |

SSRT = Stop Signal Reaction Time (in ms); RTs = Reaction Times (in ms); VRTs = Variability of Reaction Times (in ms); Accuracy = Percentage of correct responses;

^ p < 0.01 compared to Day 0;

*p < 0.05; ** p < 0.01; *** p < 0.001 compared to Day 0, anodal tDCS and sham tDCS.
